# Pulmonary Mucosa-Associated Lymphoid Tissue Lymphoma and Tuberculosis: A Rare Association With Diagnostic and Therapeutic Challenges

**DOI:** 10.14740/jmc5256

**Published:** 2026-02-02

**Authors:** Amalia Del Val Talens, Olga Balague, Sonia Rodriguez, Ana del Rio, Andrea Rivero, Pablo Mozas, Juan Correa, Eva Gine, Armando Lopez-Guillermo, Felipe Garcia, Alex Soriano, Laura Magnano

**Affiliations:** a Oncology Department, Hospital Clinic of Barcelona, Barcelona, Spain; b Pathology Department, Hospital Clinic of Barcelona, Barcelona, Spain; c Radiology Department, Hospital Clinic of Barcelona, Barcelona, Spain; d Infectious Diseases Department, Clinic Barcelona, Barcelona, Spain; e August Pi i Sunyer Biomedical Research Institute-IDIBAPS, Barcelona, Spain; f Hematology Department, Hospital Clinic of Barcelona, Barcelona, Spain; g University of Barcelona, Barcelona, Spain

**Keywords:** Pulmonary MALT lymphoma, Tuberculosis, Diagnosis, Management

## Abstract

This case report describes the rare coexistence of pulmonary tuberculosis and pulmonary mucosa-associated lymphoid tissue (MALT) lymphoma in a 68-year-old woman. The initial diagnosis of tuberculosis was supported by clinical, radiological, and microbiological findings, and the patient started on standard tuberculostatic therapy. However, the persistence of radiological abnormalities after several months of appropriate treatment, despite improvement in pleural effusion, raised suspicion for an underlying malignancy. Subsequent imaging and histopathological evaluation confirmed the diagnosis of primary pulmonary MALT lymphoma. The patient was successfully treated with immunochemotherapy, achieving complete remission. This case underscores the importance of maintaining a high index of suspicion for alternative or concomitant diagnoses, particularly malignancies, when tuberculosis exhibits an atypical clinical course or when radiological findings fail to resolve as expected. Furthermore, it highlights the need for thorough diagnostic evaluation in patients with persistent pulmonary abnormalities to ensure timely and accurate diagnosis and management.

## Introduction

Tuberculosis (TB) is one of the most prevalent infectious diseases worldwide. Although its incidence across all age groups decreased by 6–50% between 2015 and 2025, depending on the region, it remains a major public health concern, as it continues to be the leading cause of death by a single infectious agent [1, 2]. Moreover, TB is well known to be associated with an increased risk of lung cancer. In fact, it is an independent risk factor for this tumor, regardless of smoking history or chronic obstructive pulmonary disease (COPD), particularly in regions with a high incidence of TB [3]. However, its association with other malignancies, such as mucosa-associated lymphoid tissue (MALT) lymphoma, is not well established, and only a few cases have been reported in the literature. Primary pulmonary lymphoma is a rare clinical entity, accounting for less than 1% of all lymphomas. Pulmonary MALT lymphoma is the most common form, representing only 3–4% of all extranodal lymphomas [4].

Herein, we present a clinical case of a patient with the rare coexistence of TB and pulmonary MALT lymphoma. Our aim is to highlight the importance of considering alternative diagnoses when TB exhibits an atypical clinical course. Furthermore, we review all previously reported cases of this association, with a focus on the clinical, radiological, and diagnostic characteristics of pulmonary MALT lymphoma and its relationship with infections, particularly TB.

## Case Report

**Figure 1:**
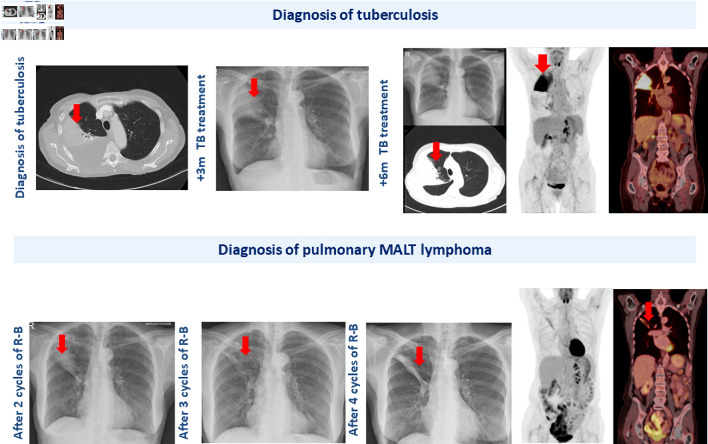
Radiological progression of the pulmonary lesion in the right upper lobe throughout the patient’s clinical course. Red arrows highlight the sequential changes observed in the pulmonary image over time.

**Figure 2:**
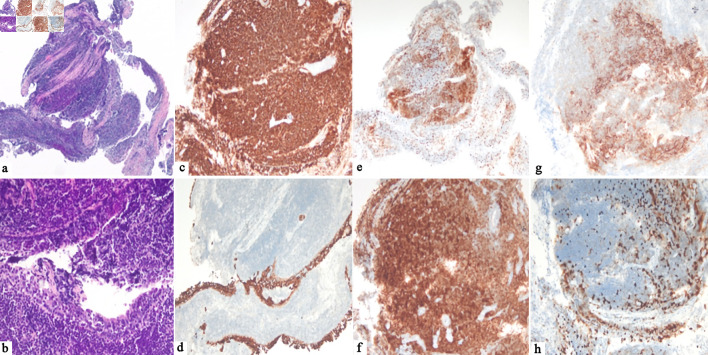
Histological feature of pulmonary MALT lymphoma. (a, b) H&E staining (× 4 and × 20) showing bronchial mucosa with an underlying infiltrate of small, mature lymphocytes with focal plasma cell differentiation. (c) Diffuse expression of CD20 (× 10) in lymphoid B cells. (d) Keratin staining (CK AE1/AE3 × 10) highlights the bronchial mucosa focally infiltrated by lymphocytes. (e) CD3 (× 4) is positive in companion lymphocytes. (f) BCL2 (× 10) is expressed in tumor cells. (g) CD21 (× 10) shows an expanded and colonized follicular dendritic cells meshwork. (h) Proliferation index (KI67, × 10) is low (10%) in the lymphoma cells. MALT: mucosa-associated lymphoid tissue; H&E: hematoxylin and eosin.

A 68-year-old woman was referred to our institution after testing positive for *Mycobacterium tuberculosis* by polymerase chain reaction (PCR) analysis of a bronchoalveolar lavage sample obtained for evaluation of a persistent cough. The patient was born and has lived her entire life in Barcelona, Spain, a low-incidence TB setting. She had a history of being an ex-smoker for 6 years (16 pack-years), a recognized risk factor for TB, but no other known illnesses or risk factors, and no history of contact with individuals with active TB. She reported fatigue and persistent, irritating cough for the previous 2 months. Auscultation revealed a decreased air entry in the upper right lobe. Blood tests showed normal leukocytes count and C-reactive protein levels. Chest radiography revealed consolidation in the upper right lobe accompanied by light pleural effusion. Thoracentesis was not performed due to the minimal volume. The bronchoalveolar lavage sample was also submitted for both acid-fast bacilli (AFB) staining and mycobacterial culture with an AFB stain positive for *Mycobacterium tuberculosis* showing a low bacillary burden. Based on these findings, a diagnosis of TB was made, and the patient started tuberculostatic treatment consisting of ethambutol, isoniazid, pyrazinamide, and rifampicin. Later, the result of culture was positive for *Mycobacterium tuberculosis*. Drug susceptibility testing was performed and demonstrated susceptibility to first-line antituberculous drugs, including rifampicin. However, after 5 months of consistent adherence to treatment, the radiological findings remained largely unchanged; consequently, a chest computed tomography (CT) scan was performed (Fig. 1). CT imaging confirmed a persistent consolidation in the right upper lobe. A positron emission tomography/computed tomography (PET/CT) demonstrated hypermetabolism (maximum standardized uptake value (SUVmax) 16.31) in the right upper lobe consolidation, raising suspicion for either an infectious or neoplastic process. Other causes, including drug-resistant TB, source control issues, and co-infections, appeared less likely in this case. Given the concern for primary pulmonary malignancy, a new bronchoscopy with bronchoalveolar lavage was performed. PCR testing for *Mycobacterium tuberculosis* was negative, and cultures for fungi and bacteria also yielded negative results. Consequently, a transbronchial lung biopsy was carried out, revealing an atypical lymphoid infiltrate by small mature B lymphocytes with focal plasma cell differentiation. These cells show diffuse CD20, CD79 and BCL2 expressions, low proliferative index (10%), and admixed CD3-positive companion T cells. CD21 immunostaining demonstrated an expanded and colonized follicular dendritic cell meshwork. Immunohistochemistry staining for BCL6, CD10, LEF1, cyclin D1, CD43, and SOX11 (Fig. 2) were negative. The morphological and immunohistochemical findings were consistent with extranodal marginal zone lymphoma of MALT lymphoma. A monoclonal immunoglobulin (IG) rearrangement in FR3 was identified, supporting the lymphoma diagnosis. Further staging studies confirmed that the disease was localized to the lung. Six months after the TB diagnosis, immunochemotherapy with rituximab and bendamustine (R-B) was initiated. Follow-up chest radiographs after each treatment cycle demonstrated progressive resolution of the pulmonary consolidation, and PET/CT imaging after four cycles confirmed complete remission (Fig. 1). The patient completed a total of 9 months of antituberculous treatment with Rifinah® (rifampicin and isoniazid) during the diagnostic workup and treatment for MALT lymphoma. Two years after completing four cycles of R-B therapy, the patient remains in complete remission, with no evidence of TB reactivation.

## Discussion

TB is one of the most prevalent infectious diseases worldwide, exhibiting a variety of radiological patterns depending on whether it is in its primary and post-primary form. Post-primary TB, which results from the reactivation of latent infection, is more commonly seen in immunocompetent adults and is typically presented as apical or subapical infiltrates or consolidations, more often in the upper lobes. Other common radiological findings include cavitations with thick walls and air-fluid levels observed in 40–87% of cases. These cavitations result from liquefied caseous material draining into the bronchial tree, which may lead to bronchogenic dissemination, radiologically identified in 19–58% of cases and in up to 98% on CT scans. Extensive cavitary disease can also lead to pleural empyema, seen as a loculated pleural collection [4, 5]. Additional features include bronchiectasis in fibrotic areas, pleural thickening or fibrosis, and bilateral involvement in advanced stages. Our patient was initially presented with pulmonary imaging suggestive of TB, supported by a positive PCR test for *Mycobacterium tuberculosis*. However, the lack of radiological improvement after 6 months of appropriate treatment, combined with the absence of evidence of ongoing infection, prompted consideration of alternative etiologies, including malignancy.

The association between lung cancer and TB is well recognized in the literature. The history of prior TB infection is considered a risk factor for lung cancer, independent of smoking and COPD [3]. The incidence of lung cancer is approximately twice as high in individuals with a history of TB compared to the general population, particularly among heavy smokers [3, 6, 7]. In contrast, the relationship between TB and other types of malignancies is less clearly established and remains incompletely understood. A meta-analysis by Leung et al [8], which aimed to quantify the global incidence of cancer attributable to TB and assess the potential benefits of TB eradication in reducing the global cancer burden, found that TB was associated with an increased risk of several malignancies, including non-Hodgkin lymphomas. Therefore, maintaining a high index of suspicion is crucial when radiological findings persist despite appropriate tuberculostatic treatment.

Pulmonary MALT extranodal marginal zone lymphoma typically affects individuals between the ages of 50 and 60 and follows an indolent course, remaining asymptomatic in approximately 50% of cases [9]. When symptoms are present, they are often nonspecific and may include cough, hemoptysis, chest pain, and dyspnea, as observed in our patient [10]. Radiologically, this lymphoma can present in four patterns: pneumonia-like consolidation, solitary nodule or mass, ground-glass opacities, and a diffuse interstitial pattern. These findings can easily mimic other conditions, such as chronic infection or a solid tumor [11]. A definitive diagnosis of pulmonary MALT lymphoma requires histopathological confirmation.

Although most cases are localized and follow an indolent course, transformation into an aggressive B-cell lymphoma could occur. Earlier studies by Zucca et al reported an estimated 5-year overall survival rate of 90% [12]. However, the optimal treatment strategy remains undefined, particularly for patients who are asymptomatic at diagnosis. Troch et al have suggested a watchful waiting approach in such cases [13]. Currently, there are no international guidelines to standardize therapeutic management. In our case, the patient was treated with immunochemotherapy due to her symptomatic presentation and the large size of the pulmonary lesion.

The underlying pathophysiology remains poorly understood. While a definitive causal relationship between TB and pulmonary MALT lymphoma cannot be established, this case allows us to hypothesize that, as in other types of MALT lymphomas, an infectious process, in this case TB, may contribute to lymphomagenesis through chronic antigenic stimulation. TB may trigger immunological activation of antigen-presenting cells, epithelial lymphocytes, B cells, and naive T cells within resting bronchus-associated lymphoid tissue (BALT), leading to sustained B-cell proliferation and differentiation. Persistent antigenic stimulation may initially result in non-neoplastic lymphoproliferative processes that can eventually progress to neoplastic forms, such as malignant lymphoma [7]. Conversely, although pulmonary MALT lymphoma is typically indolent, its presence could theoretically modulate host immune responses and facilitate TB reactivation. Which of these mechanisms best explains the coexistence of both pathologies in our case remains uncertain.

In support of this complex and potentially bidirectional association, a study by Li et al [14] in 2021 investigated factors associated with lymphoma in patients with a history of *Mycobacterium tuberculosis* infection, identifying older age and male sex as significant risk factors. The authors also reported an increased incidence of TB among lymphoma patients, particularly non-Hodgkin B lymphoma (14.6%), followed by Hodgkin (13%) and T/ natural killer (NK) lymphoma patients (11.9%) [14]. Although these findings do not establish causality, they reinforce the concept of a close interplay between TB and malignancies. In our case, it is therefore plausible that TB acted as a chronic antigenic stimulus contributing to the development of pulmonary MALT lymphoma.

In addition, the differential diagnosis should also include other lymphomas with extranodal involvement. Rare extranodal B-cell lymphomas often present a significant diagnostic challenge due to their atypical clinical and radiological presentations, which may mimic infectious processes or other non-hematologic malignancies. Several case series and reports have highlighted that involvement of unusual sites, such as the central nervous system, soft tissues, heart, adrenal glands, or thyroid, requires a comprehensive diagnostic approach integrating morphology, immunohistochemistry, extended immunophenotyping, and, when appropriate, molecular studies. Distinguishing extranodal lymphomas from chronic inflammatory conditions at certain anatomical sites can be particularly challenging, underscoring the importance of identifying clonal B-cell populations to achieve accurate classification of rare extranodal B-cell neoplasms [15, 16].

Finally, we reviewed the cases reported in the literature. We found six cases published (Table 1) [4, 17–21]. Our case represents a rare instance of pulmonary MALT lymphoma occurring concurrently with microbiologically confirmed *Mycobacterium tuberculosis* infection, distinguishing it from the most previously published. Among the seven cases reviewed, only two others (Inoue et al [17] and Inadome et al [18]) documented active TB at the time of lymphoma diagnosis. However, in both instances, full clinical details were limited to abstracts. In contrast, the remaining cases either reported a history of past TB infection or no documented evidence of TB at all [19–21]. Notably, our patient exhibited typical clinical features of pulmonary MALT lymphoma, consistent with previously published case reports [4, 17–21]. A paucisymptomatic presentation in a patient aged 50–60 years with lung consolidation is considered the most common clinical scenario for pulmonary MALT lymphoma [4, 9], often contributing to an incorrect or delayed diagnosis. Moreover, unlike most previously reported patients who often had either empirical TB treatment or prior infections, our case highlights the diagnostic challenges posed by overlapping clinical and radiological features of TB and lymphoma, emphasizing the need for high suspicion and tissue diagnosis in non-resolving cases.

**Table 1:** Summary of Reported Cases of Pulmonary MALT Lymphoma in Association With Tuberculosis

Case report	Sex	Age	Symptoms	Confirmed TB at diagnosis/past	Treated TB at diagnosis of lymphoma
Our case	F	68	Shortness of breath, cough	Yes	Yes
Kou et al, 2023 [4]	F	46	Fever, cough, shortness of breath, persistent right lung consolidation	No/no	Yes
Gu et al, 2023 [19]	M	66	Cough, expectoration, dyspnea	No/yes (5 years ago)	No
Yang et al, 2020 [20]	M	65	Increased right upper lobe shadow after 9-month anti-TB treatment	No/yes	No
Magazine et al, 2014 [21]	M	53	Chronic cough, exertional breathlessness, intermittent fever, weight loss and anorexia	No/yes (5 years ago)	No
Inoue et al, 2012 [17]	F	66	Right upper lobe mass in X-ray examination	Yes	Not available
Inadome et al, 2001 [18]	M	70	Not mentioned	Yes	Not mentioned

MALT: mucosa-associated lymphoid tissue; TB: tuberculosis; F: female; M: male.

This case underscores the diagnostic challenges encountered when *Mycobacterium tuberculosis* infection coexists with other pathologies, particularly malignancies. The significant clinical and radiological overlap between TB and conditions such as MALT lymphoma can lead to delays or misdiagnosis, especially when TB is microbiologically confirmed. Consequently, it is essential to maintain a high index of suspicion for underlying malignancy in cases with persistent or atypical findings. Early tissue sampling remains a critical step to ensure timely diagnosis and appropriate treatment of concurrent diseases.

### Learning points

This case highlights the importance of maintaining a high index of suspicion for malignancy in patients with persistent pulmonary abnormalities, even when TB is microbiologically confirmed. The coexistence of pulmonary MALT lymphoma and TB can obscure diagnosis and delay appropriate treatment. Early histopathological evaluation is essential in atypical or non-resolving cases to ensure accurate diagnosis and timely management.

## Data Availability

The authors declare that data supporting the findings of this study are available within the article.

## Abbreviations

TB: tuberculosis
MALT: mucosa-associated lymphoid tissue
COPD: chronic obstructive pulmonary disease
CT: computed tomography
PET: positron emission tomography
PET/CT: positron emission tomography/computed tomography
PCR: polymerase chain reaction
BALT: bronchus-associated lymphoid tissue
